# synDNA—a Synthetic DNA Spike-in Method for Absolute Quantification of Shotgun Metagenomic Sequencing

**DOI:** 10.1128/msystems.00447-22

**Published:** 2022-11-01

**Authors:** Livia S. Zaramela, Megan Tjuanta, Oriane Moyne, Maxwell Neal, Karsten Zengler

**Affiliations:** a Department of Pediatrics, University of California, San Diego, La Jolla, California, USA; b Center for Microbiome Innovation, University of California, San Diego, La Jolla, California, USA; c Department of Bioengineering, University of California, San Diego, La Jolla, California, USA; Luxembourg Centre for Systems Biomedicine

**Keywords:** absolute abundance, metagenomics, microbial communities

## Abstract

Microbiome studies have the common goal of determining which microbial taxa are present, respond to specific conditions, or promote phenotypic changes in the host. Most of these studies rely on relative abundance measurements to drive conclusions. Inherent limitations of relative values are the inability to determine whether an individual taxon is more or less abundant and the magnitude of this change between the two samples. These limitations can be overcome by using absolute abundance quantifications, which can allow for a more complete understanding of community dynamics by measuring variations in total microbial loads. Obtaining absolute abundance measurements is still technically challenging. Here, we developed synthetic DNA (synDNA) spike-ins that enable precise and cost-effective absolute quantification of microbiome data by adding defined amounts of synDNAs to the samples. We designed 10 synDNAs with the following features: 2,000-bp length, variable GC content (26, 36, 46, 56, or 66% GC), and negligible identity to sequences found in the NCBI database. Dilution pools were generated by mixing the 10 synDNAs at different concentrations. Shotgun metagenomic sequencing showed that the pools of synDNAs with different percentages of GC efficiently reproduced the serial dilution, showing high correlation (*r* = 0.96; *R*^2^ ≥ 0.94) and significance (*P* <* *0.01). Furthermore, we demonstrated that the synDNAs can be used as DNA spike-ins to generate linear models and predict with high accuracy the absolute number of bacterial cells in complex microbial communities.

**IMPORTANCE** The synDNAs designed in this study enable accurate and reproducible measurements of absolute amount and fold changes of bacterial species in complex microbial communities. The method proposed here is versatile and promising as it can be applied to bacterial communities or genomic features like genes and operons, in addition to being easily adaptable by other research groups at a low cost. We also made the synDNAs’ sequences and the plasmids available to encourage future application of the proposed method in the study of microbial communities.

## INTRODUCTION

Metagenomics has become a well-established approach for profiling microbial communities and studying microbiomes in the environment, as well as in health and disease (1). Correlating shifts in the relative composition of microbial communities among different conditions to draw explanatory hypotheses is the goal of many microbiome surveys (2–4). A fundamental setback limitation of these studies is that taxon abundances are calculated consisting of only relative values (5–7). Mathematically, an increase in the relative abundance of one taxon results in a decrease in the relative abundance across the remaining taxa, even if their absolute abundance remains unchanged (8). Thus, the relative abundance of one taxon is artificially constrained to the abundance of all other taxa. Recent statistical methods acknowledge these biases and aim to address them by using the ratios among taxa, which are conserved regardless of whether the data are relative or absolute (6, 7, 9). However, these postanalytical data modifications necessitate *a priori* knowledge of the studied community to appropriately select the reference taxa used to calculate the ratios. Thus, an intrinsic limitation of relative abundance methods is that they cannot quantify the magnitude of the changes of an individual taxon in two samples or determine if this taxon is, in absolute numbers, more or less abundant.

Absolute abundance measurements, on the other hand, are less ambiguous and could be easily matched across studies, thus enabling cross-comparison and increasing reproducibility. Absolute abundance quantification is presently accessed through time-consuming, often costly or laborious experimental approaches, such as quantitative plating via colony counting, quantitative PCR (qPCR), flow cytometry, species-specific fluorescence *in situ* hybridization, or a combination of single-cell and functional-targeting methods with genomics (10–12). Another issue with these approaches is that they are currently limited to a few targets (genes or organisms) and, more importantly, require *a priori* knowledge of the microbial community composition under investigation.

Another approach is the use of exogenous spike-in bacteria (i.e., whole cells) to calculate the ratio of absolute bacterial abundances and to evaluate the structure and the dynamics of microbiomes (13). Whole-cell spike-in controls have the advantage that they have the same property as the target sample (i.e., living cells) and that the control can be used to benchmark the entire process—from sample storage, DNA extraction, and metagenomic library generation to defining thresholds and parameters for computational analysis. However, spike-in whole bacterial cells can strongly interfere with downstream analysis, especially if the chosen bacterium is part of the actual microbiota or when its genome shares similarities with other bacteria in the community under investigation. Furthermore, DNA extraction methods may yield substantial differences between species and results (14). Once again, *a priori* knowledge about community composition is thus crucial for quantification using spike-in whole cells.

One alternative to overcome these limitations is the use of nucleic acid spike-in controls with defined quantities—a practice commonly used in analytical fields. The use of spike-in standards into biological samples had been proposed a few years back for transcriptome sequencing (RNA-Seq) methods (15, 16) and has recently been extended to 16S rRNA gene sequencing (10, 17, 18). While these spike-in controls are increasing reproducibility and enabling benchmarking of experimental methods, only a few examples are available for shotgun metagenomics. Of note, Hardwick et al. (19) generated a robust method to perform absolute quantification. By using a set of multiple synthetic DNA sequences, the method allows for the capture of true mock microbial community compositions with high accuracy (19). Although this method shows great potential, it is still not highly disseminated among the scientific community due to the difficulty in sharing the sequences between laboratories. The development of a universal method that is independent of microbial composition and does not require *a priori* information is not only cost-effective, but it can also be easily disseminated among the scientific community and is therefore crucial to further improve microbiome research. Here, we describe and validate a novel absolute quantification method based on synthetic DNA (synDNA) spike-in pools for shotgun metagenomic sequencing.

## RESULTS

### Evaluation of 16S synthetic spike-ins for shotgun metagenomics.

First, we evaluated the publicly available synthetic spike-ins to determine their potential application for shotgun metagenomic experiments. Tourlousse et al. (17) developed synthetic 16S rRNA spike-in genes containing conserved regions, necessary for PCR primer amplification, and synthetic sequences randomly generated. The authors showed that the synthetic 16S rRNA spike-ins were efficiently used to assess data quality and absolute quantification in 16S rRNA sequencing experiments. Using *in silico* analysis, we evaluated the potential use of synthetic 16S rRNA spike-ins for shotgun metagenomic sequencing.

We downloaded sequencing data for 8 bacterial isolates representing 5 different species (Escherichia coli, Bacteroides vulgatus, Clostridium acetobutylicum, Gemmatimonas aurantiaca, and Treponema bryantii) used as templates to design the synthetic 16S spike-in genes (17). As expected, we observed that the reads from the 8 isolates aligned preferentially to the synthetic 16S spike-in gene designed based on the same species. For instance, Escherichia coli ASM882318v1 reads aligned preferentially to synthetic 16S spike-in genes designed upon E. coli genomes (Ec5001, Ec5002, Ec5003, Ec5004, Ec5005, Ec5501, and Ec5502) (see Fig. S1A in the supplemental material), although, random alignments were observed for all bacterial isolates: for example, reads from all isolates were aligned to the spike-in Ga5501 designed upon the *G. aurantiaca* genome (Fig. S1A). These random alignments indicate the unpredictability of using synthetic 16S spike-in genes for shotgun metagenomic sequencing. Reads from multiple species in the microbial community can be wrongly counted as a spike-in read, leading to inaccurate quantification.

10.1128/msystems.00447-22.1 FIG S1 (A) Alignment of whole bacterial genome sequencing reads to synthetic 16S spike-in genes: Escherichia coli 9001_S21 (ERR1877982), Escherichia coli ASM882318v1 (SRR10015223), Clostridium acetobutylicum RH8 (SRR1217786), Clostridium acetobutylicum ATCC 824 (SRR1700598), Treponema bryantii XBD1002 (SRR4140225), Bacteroides vulgatus UBA7045 (SRR5327338), Gemmatimonas aurantiaca MTG (SRR6485762), Bacteroides vulgatus (SRR8060827), and Treponema bryantii NK4A124 (SRR896066). (B) Reads from published shotgun metagenomic data were aligned to the synthetic 16S rRNA spike-in genes. Each dot represents one shotgun metagenomic library. Sequence data analysis was performed using standard protocols (see Materials and Methods). Download FIG S1, EPS file, 0.7 MB. Copyright © 2022 Zaramela et al. 2022 Zaramela et al. https://creativecommons.org/licenses/by/4.0/ This content is distributed under the terms of the Creative Commons Attribution 4.0 International license.

Furthermore, we demonstrated the occurrence of random read counts by computing false-positive alignments to the synthetic 16S spike-in genes using shotgun metagenomic data from different biological sources, including samples from the ocean (20, 21), soil (22), gut (23, 24), saliva (25), and skin (26). For all of these samples, reads were wrongly counted as a spike-in, showing a wide range of false-positive alignments (from 0.3× to 43× genome coverage per 1 million reads) among and within the different biological sources (Fig. S1B).

### synDNA design and performance.

**FIG 1:**
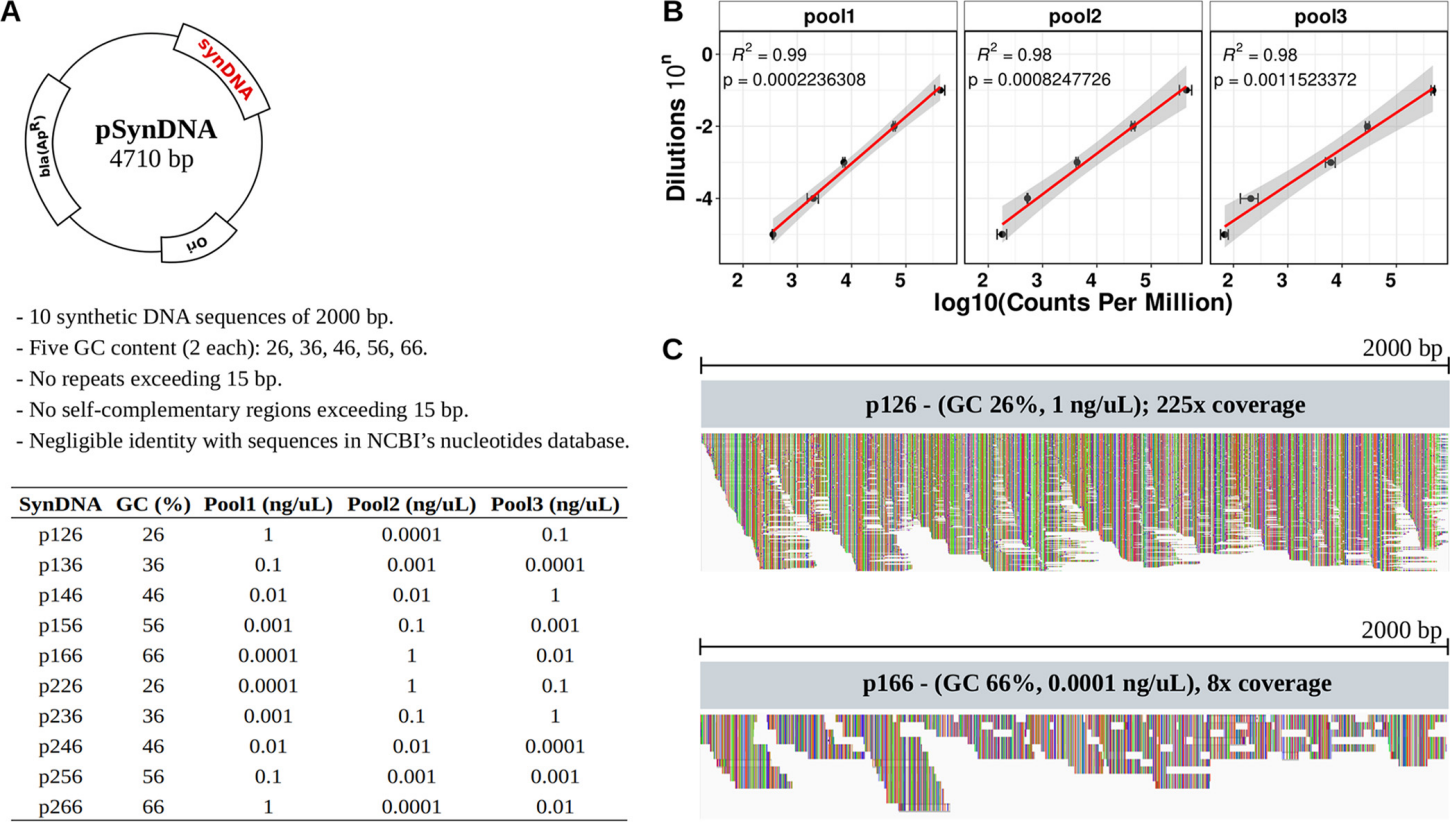
Overview of synDNA design and sequencing performance. (A) synDNA design and synthesis. Ten synthetic DNA (synDNA) sequences with various percentages of GC were computationally generated and sent for *de novo* synthesis. The sequences were cloned to the plasmid pUC57 for easy sharing between laboratories. Serial dilutions were used to generate different synDNA pools; the concentrations of the different plasmids were varied. (B) Representative examples of the serial dilutions were used to generate calibration curves for absolute quantification. Error bars indicate SD between replicates. The full panel of dilutions including all pools and replicates is shown in Fig. S5. (C) Genome browser views of two synDNAs (p126, GC, 26%, 1-ng/μL dilution; p166, GC, 66%, 0.0001-ng/μL dilution) demonstrate the dilutions are adequate to provide sufficient coverage to all synDNA sequence extension.

To circumvent the limitations of the currently available methods, we computationally designed a set of 10 synthetic DNAs (synDNAs) with negligible similarity to sequences present in the NCBI Nucleotide database. In addition, to minimize the PCR amplification bias associated with GC-rich and AT-rich reads (27), synDNAs were synthesized to cover a wide range of GC content (26 to 66%). The 10 synDNAs were cloned into the E. coli plasmid pUC57, which can be easily obtained from Addgene (see Materials and Methods), maintained, and distributed among laboratories (Fig. 1A).

We further designed qPCR primers to assess the concentration of synDNAs across different samples and dilutions. To create a calibration curve, the 10 synDNAs were serially diluted, ranging in concentration from 10^−4^ ng/μL to 100 ng/μL, and the serial dilution accuracy was evaluated by qPCR (Fig. S2). Similar to the analysis performed for the synthetic 16S rRNA genes (Fig. S1A and B), the synDNAs’ sequences were used as reference to align reads from 436 shotgun metagenomic experiments (20–26). No alignment (0%) was observed for sequence data of different biological sources (e.g., from ocean, soil, gut, saliva, or skin). These results reinforce the efficacy of the computational approach used to generate our artificial DNA sequences to avoid nonspecific alignment with natural microbial genomes.

10.1128/msystems.00447-22.2 FIG S2 The 10 synDNAs were serially diluted, ranging in concentrations from 10^−4^ ng/μL to 100 ng/μL. qPCRs were performed to check the accuracy of the plasmid dilutions before generating the synDNA pools. All qPCRs were performed in triplicate. Data points were fitted to a linear model (dilution 10*^n^* ~ log_10_ cpm). *R*^2^ represents the coefficient of determination and p the *P value*. Bars around the dots represent the standard error among the triplicates. Download FIG S2, EPS file, 0.6 MB. Copyright © 2022 Zaramela et al. 2022 Zaramela et al. https://creativecommons.org/licenses/by/4.0/ This content is distributed under the terms of the Creative Commons Attribution 4.0 International license.

### Sequencing of synDNA pools.

Next, we evaluated if the synDNA concentrations maintain a linear relationship when pooled for DNA sequencing experiments. The 10 synDNAs were combined in three pools with various concentrations of the individual plasmids, taking into account the different percentages of GC in order to minimize the bias associated with GC-rich and AT-rich regions (Fig. 1A).

The pools were sequenced in triplicate. We evaluated the distribution of sequencing errors across the 10 synDNAs in the 3 pools (9 libraries). Similar to previous publications (28, 29), we observed the sequencing errors and GC content followed a quadratic polynomial model, with errors prevalently associated with GC-poor sequences (<40%) (Fig. S3). Browne et al. (28) demonstrated that the sequencing error using Illumina platforms increased severely outside of the GC range of 45 to 65%, which corroborates with our findings of 26% and 36% GC reads showing a higher base call error rate (Fig. S3). Despite the sequencing errors, all pools’ dilutions showed a high coefficient of determination (*R*^2^ ≥ 0.94) and high significance (*P* < 0.01) between dilution and sequencing reads (in counts per million) (Fig. 1B), as well as excellent reproducibility between technical replicates (Fig. S4). Pool 1 presented the highest coefficient of determination (*R*^2^ ≥ 0.99), followed by pool 3 (*R*^2^ ≥ 0.97) and pool 2 (*R*^2^ ≥ 0.94). Overall, these results validate the approach of pooling synDNAs with different percentages of GC content to evaluate the linear relationship between synDNA concentration and sequencing data counts.

10.1128/msystems.00447-22.3 FIG S3 Error call for shotgun metagenomic sequencing across 3 different synDNA pools. Data points were fitted to a quadratic model. *R*^2^ represents the coefficient of determination and p the *P* value. Bars around the dots represent the standard error among the triplicates. Download FIG S3, EPS file, 0.9 MB. Copyright © 2022 Zaramela et al. 2022 Zaramela et al. https://creativecommons.org/licenses/by/4.0/ This content is distributed under the terms of the Creative Commons Attribution 4.0 International license.

10.1128/msystems.00447-22.4 FIG S4 Shotgun metagenomic sequencing of synDNA pools. Data points were fitted to a linear model (dilution 10*^n^* ~ log_10_ CPM). *R*^2^ represents the coefficient of determination and p the *P* value. Bars around the dots represent the standard error among the triplicates. Download FIG S4, EPS file, 0.5 MB. Copyright © 2022 Zaramela et al. 2022 Zaramela et al. https://creativecommons.org/licenses/by/4.0/ This content is distributed under the terms of the Creative Commons Attribution 4.0 International license.

### Using synDNA pools to predict absolute abundances in mock communities.

We evaluated if the synDNAs could be used to estimate the absolute abundance of organisms in a microbial community. We combined the synDNA pools (pools 1, 2, and 3) with a commercial mock community containing 8 bacterial species (i.e., Bacillus subtilis, Enterococcus faecalis, Escherichia coli, Lactobacillus fermentum, Listeria monocytogenes, Pseudomonas aeruginosa, Salmonella enterica, and Staphylococcus aureus) with a known composition (ZymoBIOMICS Microbial Community DNA). We observed that the linear relationship among the synDNAs and their dilutions were maintained, with pools 1 and 3 showing high coefficients of determination (*R*^2^ ≥ 0.96) and being highly significant (*P* <* *0.01) (Fig. S5). Pool 2 showed moderated coefficients of determination (0.67 ≤ *R*^2^ ≥ 0.86) due to potential pipetting inaccuracies while mixing the synDNAs to create pool 2 (Fig. 1 and Fig. S5). Despite the observation of reverse reads presenting poor quality compared to forward reads described in the literature (30), we did not observe any specific bias associated with forward or reverse reads, allowing us to combine them to generate a unique linear model per sample.

10.1128/msystems.00447-22.5 FIG S5 The 10 synDNAs were serially diluted in concentrations from 10^−4^ ng/μL to 1 ng/μL (Fig. 1). Pools 1 and 3 were mixed with ZymoBIOMICS Microbial Community DNA in biological replicates (two vials), and the sequencing was performed in three technical replicates. Data points were fitted to a linear model (dilution 10*^n^* ~ log_10_ CPM). *R*^2^ represents the coefficient of determination and p the *P* value. Bars around the dots represent the standard error among the triplicates. Download FIG S5, EPS file, 1.1 MB. Copyright © 2022 Zaramela et al. 2022 Zaramela et al. https://creativecommons.org/licenses/by/4.0/ This content is distributed under the terms of the Creative Commons Attribution 4.0 International license.

To evaluate if the synDNA pools could be used to predict the absolute number of cells, we combined the synDNAs of one synDNA pool (pool 1, 2, or 3) into an individual sample. We used the total number of reads aligned to each synDNA sequence and the synDNA’s dilution to obtain a linear model for each experiment. The linear models were used to estimate the total amount of genome copies per organism in the community. Prokaryotic organisms (e.g., bacteria) usually have only one copy of their chromosomes (also known as haploid) (31). However, the number of genome copies per bacterial cell depends on the growth rate. As a simplified approach, we considered that each cell contains one genome copy. In order to compare our predictions with the manufacturer’s information, we calculated the absolute number of genome copies (or cells per organism) for each of the bacteria in the mock community (see the manufacturer’s information and Material and Methods). We then translated this absolute number into a percentage in order to compare the measured percentage to the expected percentage provided by the manufacturer. We additionally compared the percentage of cells per organism to the relative abundance of the species, which was calculated based on the sequencing read counts, as commonly used.

**FIG 2:**
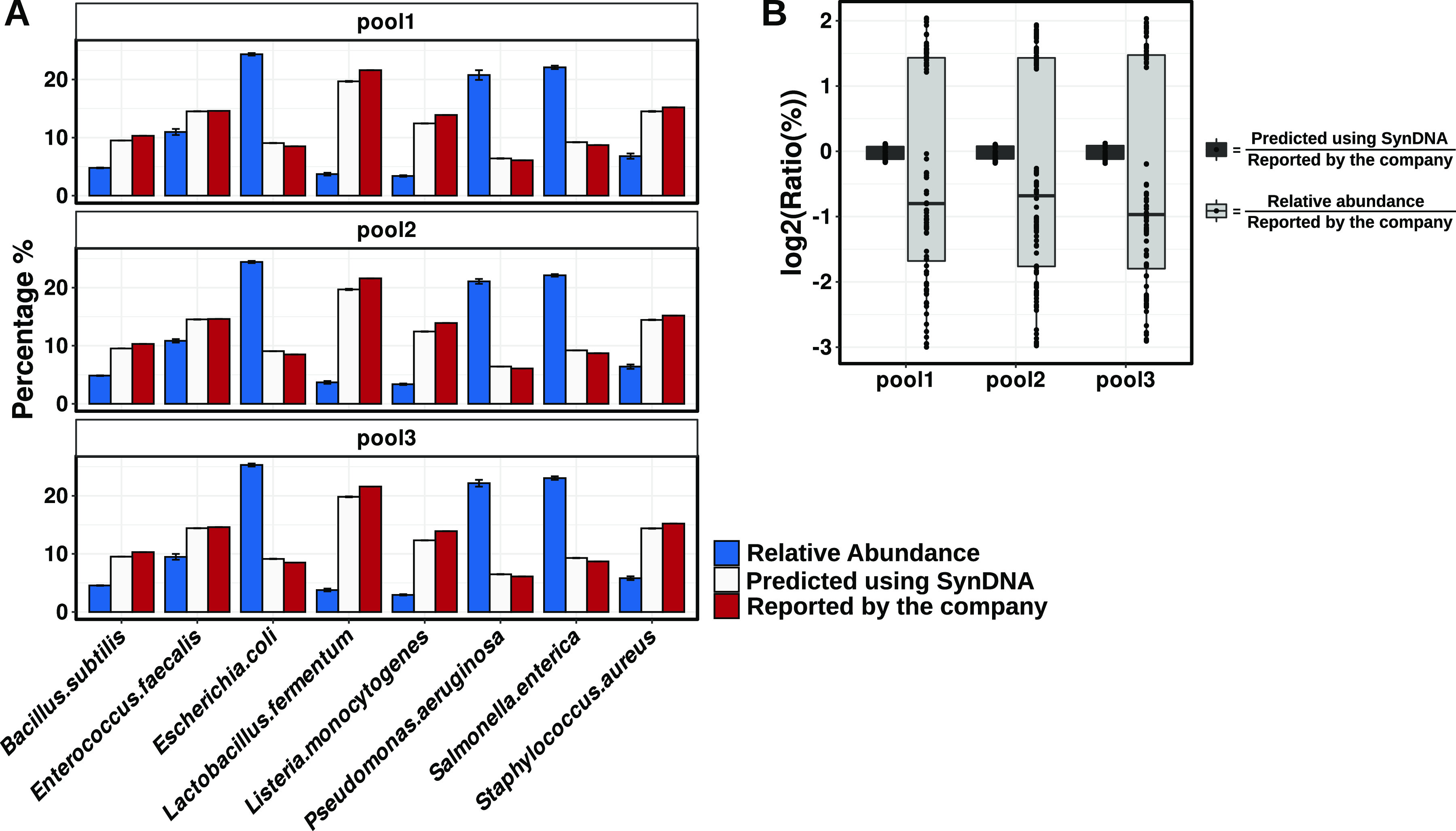
Comparison among percentages of genome copy per bacterial species in ZymoBIOMICS Microbial Community DNA across different quantification methods. (A) Percentage of cells and reads per bacterial species. Blue bars, percentage of the number of genome copies obtained by relative read count analysis; white bars, percentage of the number of genome copies or cells predicted using the linear models generated by the synDNAs; red bars, percentage of the number of genome copies or cells reported in the commercial ZymoBIOMICS Microbial Community DNA. (B) Log ratio between predicted and expected numbers of genome copy per bacterial species in the ZymoBIOMICS Microbial Community DNA.

We observed that the linear models generated using the synDNAs were able to predict the percentage of each bacteria species in the commercial mock community with high accuracy, while the relative abundance based on read counts poorly reproduced the real microbial community composition (Fig. 2A and B). The ratios between the measured values and the expected values were centered around zero, showing small variation between the expected and the measured values. On the other hand, when using the relative abundance based on read counts, we observed large dispersion of the log ratios around zero (Fig. 2B). Overall, these data show that our approach allows us to estimate the composition of the microbial community with high accuracy and reproducibility compared to commonly used relative abundance methods based on normalized read counts.

For all pools, the predicted (using synDNA) and the reported (manufacturer’s report) percentages of absolute number of cells were highly correlated (*r* > 0.99; *P* <* *0.00001) (Table 1). Compared to the relative abundance, the correlation between the reported values and the calculated ones is negative (*r* = −0.749), which indicates that the opposite conclusion would be obtained using relative abundance as the method of analysis. For example, in the mock community, 21.6% of the total number of cells are from *L. fermentum* and 8.5% are from E. coli, which means 2.5× more *L. fermentum* than E. coli cells. The relative abundance analysis showed the opposite result, with *L. fermentum* representing only 3.7% of the normalized reads and E. coli 24.3%. Our synDNA method accurately estimated the expected ratio by predicting 2.2× more cells of *L. fermentum* than E. coli.

**TABLE 1:** synDNA linear model performance in predicting the mock community composition

Species	GC content (%)	Mock (%)	Pool 1^*a*^			Pool 2^*b*^			Pool 3^*c*^			Relative abundance^*d*^		
			Predicted (%)	SD (%)	Error (%)	Predicted (%)	SD (%)	Error (%)	Predicted (%)	SD (%)	Error (%)	Predicted (%)	SD (%)	Error (%)
*B. subtilis*	43.9	10.3	9.508	0.017	7.69	9.526	0.011	8.13	9.511	0.017	7.66	4.788	0.063	89.09
*E. faecalis*	37.5	14.6	14.532	0.027	0.47	14.529	0.023	0.49	14.420	0.030	1.23	10.967	0.522	70.76
*E. coli*	46.7	8.5	9.068	0.030	6.69	9.065	0.016	6.23	9.142	0.029	7.56	24.351	0.218	47.86
*L. fermentum*	52.4	21.6	19.671	0.081	8.93	19.689	0.116	9.71	19.806	0.076	8.31	3.709	0.243	92.92
*L. monocytogenes*	38	13.9	12.438	0.023	10.52	12.446	0.040	11.68	12.330	0.022	11.30	3.389	0.231	91.08
*P. aeruginosa*	66.2	6.1	6.422	0.037	5.28	6.429	0.017	5.12	6.491	0.030	6.41	20.765	0.837	68.63
*S. enterica*	52.2	8.7	9.215	0.034	5.91	9.211	0.018	5.55	9.293	0.034	6.81	22.083	0.297	57.70
*S. aureus*	32.9	15.2	14.511	0.054	4.53	14.448	0.061	5.21	14.386	0.042	5.36	6.812	0.447	79.30

a Correlation test: *P* = 0.9920, *P* value = 1.242e206.

b Correlation test: *P* = 0.9923, *P* value = 1.12e206.

c Correlation test: *P* = 0.9916, *P* value = 1.486e206.

d Correlation test: *P* = 20.749, *P* value = 0.0325.

In terms of percentage of error, our linear models showed a range of 0.47 to 11.68% prediction error, while that for the relative abundance showed a range of 47.86% to 92.92% (Table 1). The percentage of error associated with L. monocytogenes cannot be explained by either percentage of GC or genome length. L. monocytogenes has a GC content of 38% and a genome length of 2.992 Mbp, similar to those of E. faecalis with 37.5% and 2.845 Mbp, which did not exhibit a high prediction error rate. In addition, we looked at the sequencing error in the reads that aligned to the genomes of *L. fermentum*, L. monocytogenes and E. faecalis. All three species presented very similar error distributions, with average values of 2.85, 2.63, and 2.59%, respectively (Fig. S6). Thus, these errors might be associated with intrinsic limitations of the sequencing and alignment methods and also might be due to the impossibility to synchronize growth rates in microbial communities, which can lead to more than one genome copy per cell as discussed before. However, all of the limitations of the proposed approach do not jeopardize the robustness of our quantification method. Overall, these results show that using our synDNAs pools as spike-ins allows the accurate measurement of absolute abundances within a bacterial community.

10.1128/msystems.00447-22.6 FIG S6 Sequencing error distribution for reads that aligned to the genomes of Lactobacillus fermentum, Listeria monocytogenes, and Enterococcus faecalis. Download FIG S6, EPS file, 0.1 MB. Copyright © 2022 Zaramela et al. 2022 Zaramela et al. https://creativecommons.org/licenses/by/4.0/ This content is distributed under the terms of the Creative Commons Attribution 4.0 International license.

### Using synDNA pools to predict absolute abundances in complex microbial communities.

**FIG 3:**
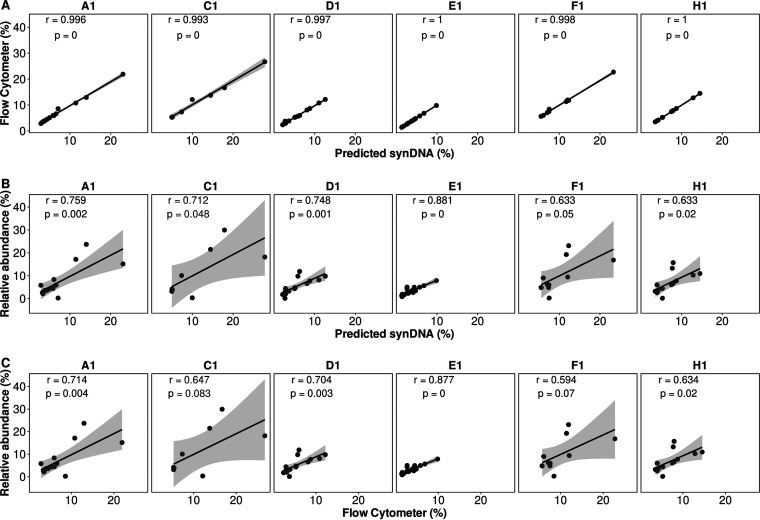
Pearson correlation analysis. Correlation between predicted percentage of microbial species using synDNA, read counts normalized by flow cytometer cell counts, and traditional relative abundance. (A) Correlation between predicted percentage of microbial species using synDNA and read counts normalized by flow cytometer cell counts; (B) correlation between predicted percentage of microbial species using synDNA and traditional relative abundance; (C) correlation between predicted percentage of microbial species using read counts normalized by flow cytometer cell counts and traditional relative abundance. Predicted percentage was calculated by applying the synDNA linear models obtained using pool 2 and forward reads. The same analysis using pools 1 and 3 is described in the Table S1. A figure including Prevotella melaninogenica is available as Fig. S8A to C. To improve reproducibility, only microbe species with more than 1% genome coverage were included in the analysis (*n* = 24). For comparison purposes, the analysis including all rare/low abundant taxa (*n* = 930) is available as Fig. S8D to F. A1, C1, D1, E1, F1, and H1 indicate the saliva samples obtained from 6 different individuals.

Finally, we evaluated the synDNA performance in complex microbial communities. We spiked the synDNA pools (pools 1, 2, and 3) (Fig. 1) into DNA extracted from human saliva that was previously quantified using a flow cytometer (32). Given the intrinsic sparsity of microbiome data (33) and that many rare or very low abundant taxa are caused by sequencing artifacts, contamination, and/or sequencing errors (33), only microbe species with more than 1% genome coverage were included in the analysis. The linear models per sample were obtained as described in the Materials and Methods section (Fig. S7). The total number of microbial species cells in each saliva sample was used to normalize the sequencing read counts and to compare with the synDNA prediction (Fig. 3). We also compared it with the relative abundance normalization method, in which the total number of reads aligned to one taxon is normalized by the total number of reads sequenced per sample. Results of correlation analysis between the predicted percentage of microbial species using synDNA and read counts normalized by flow cytometer cell counts were significant and above 0.99 for all samples (Fig. 3A). On the other hand, comparison of commonly used relative abundance with synDNA and flow cytometry predictions showed inconsistent results, with correlation coefficients as low as 0.6 for half of the samples (samples C1, F1, and H1) (Fig. 3B and C). The unexpected higher correlation coefficient observed for sample E1 is mostly driven by the top right (Prevotella melaninogenica) influential point (Fig. S8A to C). After outlier removal, the correlation coefficients drop to 0.881 and 0.877 for comparisons of relative abundance versus predicted synDNA or relative abundance versus flow cytometer, respectively.

10.1128/msystems.00447-22.7 FIG S7 The 10 synDNAs were serially diluted in concentrations from 10^−4^ ng/μL to 1 ng/μL (Fig. 1). Pools 1, 2, and 3 were mixed with saliva DNA from 6 individuals (A1 to H1). Data points were fitted to a linear model (dilution 10*^n^* ~ log_10_ CPM). *R*^2^ represents the coefficient of determination and p the *P* value. Download FIG S7, EPS file, 1.0 MB. Copyright © 2022 Zaramela et al. 2022 Zaramela et al. https://creativecommons.org/licenses/by/4.0/ This content is distributed under the terms of the Creative Commons Attribution 4.0 International license.

10.1128/msystems.00447-22.8 FIG S8 (A to C) Pearson correlation analysis including Prevotella melaninogenica (*n* = 25). Shown are the results from correlation analysis between the predicted percentage of microbial species using synDNA, read counts normalized by flow cytometer cell counts, and traditional relative abundance. (A) Correlation between predicted percentage of microbial species using synDNA and read counts normalized by flow cytometer cell counts; (B) correlation between predicted percentage of microbial species using synDNA and traditional relative abundance; (C) correlation between predicted percentage of microbial species using read counts normalized by flow cytometer cell counts and traditional relative abundance. Only microbes with more than 1% genome coverage were included in the analysis. The predicted percentage was calculated by applying the synDNA linear models obtained using pool 2 and forward reads. (D to F) Pearson correlation analysis including all rare/low-abundance taxa (*n* = 930). Shown are the results from correlation analysis between predicted percentage of microbial species using synDNA, read counts normalized by flow cytometer cell counts, and traditional relative abundance. (D) Correlation between predicted percentage of microbial species using synDNA and read counts normalized by flow cytometer cell counts; (E) correlation between predicted percentage of microbial species using synDNA and traditional relative abundance; (F) correlation between predicted percentage of microbial species using read counts normalized by flow cytometer cell counts and traditional relative abundance. Only microbes with more than 1% genome coverage were included in the analysis. Predicted percentage was calculated by applying the synDNA linear models obtained using pool 2 and forward reads. Download FIG S8, PDF file, 0.3 MB. Copyright © 2022 Zaramela et al. 2022 Zaramela et al. https://creativecommons.org/licenses/by/4.0/ This content is distributed under the terms of the Creative Commons Attribution 4.0 International license.

10.1128/msystems.00447-22.9 TABLE S1 Correlation analysis for the synDNA pools spiked into the saliva samples. Download Table S1, XLSX file, 0.05 MB. Copyright © 2022 Zaramela et al. 2022 Zaramela et al. https://creativecommons.org/licenses/by/4.0/ This content is distributed under the terms of the Creative Commons Attribution 4.0 International license.

**FIG 4:**
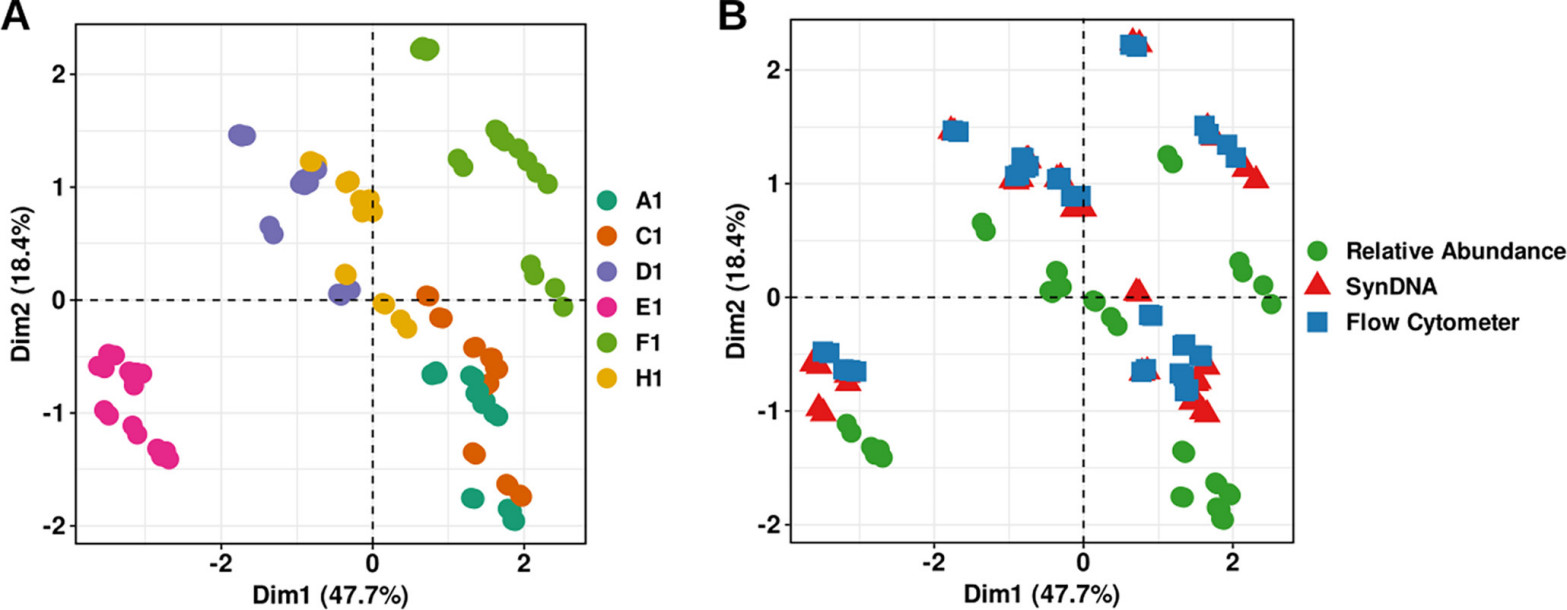
Principal-component analysis. Shown are percentages of microbial species clustered by participants in PCA space using Euclidean distance. (A) Samples are colored by participants; (B) samples are colored by normalization method.

The synDNA pools (pools 1, 2, and 3) (Fig. 1) also exhibited small differences in performance (Table S1), in which pools 1 and 2 presented the best outcome. Additionally, in our principal-component analyses (PCA), the samples clustered more by participant (Fig. 4A) than by normalization method (Fig. 4B).

However, the microbial abundance predicted using synDNA models and normalized by flow cytometer cell counts clustered tightly, independent of the synDNA pool spiked into the sample (Fig. 4). Taken together, these results confirm the applicability of synDNAs to obtain absolute abundance of microbes in complex samples, such as saliva, that are very dependent on the subject, present high contamination with human DNA, and show low representativity in reference databases.

## DISCUSSION

Shotgun metagenomics has greatly contributed to our comprehension of microbial communities’ composition, functionality, and dynamics. However, analysis and comparison of different experiments between different research groups are challenging due the lack of reproducibility and standardization (34). Most metagenomic studies rely on relative abundance to drive their conclusions (3, 4, 25, 26). Despite all of the computational and mathematical methods created to minimize the bias associated with relative measurements, internal references would immensely contribute to generation of a standardized protocol and allow for the calculation and comparison of absolute abundance between samples. Here we describe the development of synDNAs, synthetic DNA sequences covering diverse GC contents and showing negligible similarity to natural DNA sequences. When added to the DNA samples before library preparation, the synDNAS can be used to evaluate library quality, to calculate the sequencing error rate, to assess technical variation between samples, and to calculate the absolute abundance of bacterial species. Additionally, synDNAs can be used in combination with statistical methods that use ratios to minimize relative abundance biases (6, 7, 9). These methods require the choice of one common microbial taxon to be the ratio's denominator across samples. Although the different methods present guidelines, including mathematical models, on how to perform this choice, choosing a particular taxon is still arbitrary. The synDNA spike-ins offer an attractive addition to these methods, since the synDNAs’ concentrations will be kept unchanged across samples, providing a uniform denominator.

Our results indicate that the synDNAs allowed the absolute quantification of the total number of bacterial cells with high reproducibility and accuracy. The design of synDNAs comprising a range of GC contents minimized analysis bias and enabled the generation of accurate prediction models. Finally, synDNAs are easily distributed into E. coli plasmids and are readily available to the scientific community at Addgene (see Materials and Methods). We validated our findings using a commercial microbial mock community with a known amount of cells per bacterial species. Additionally, we applied the synDNA to complex DNA samples extracted from human saliva samples. In all of the cases, the synDNA approach outperformed relative abundance measurements and proved to be a straightforward method to be applied in studies of microbial communities.

## MATERIALS AND METHODS

### Computational simulation: sequence definition and bias prediction.

The synthetic DNA genes (synDNAs) were computationally generated using R scripts. In brief, we used the package Biostrings to create a function called “*generateSeqs*” that randomly draws one of the four nucleotides at a time (A, T, G, and C), respecting the proportion (GC content) predetermined by the user. The R script used to generate the synDNAs' sequences is available in GitHub (https://github.com/lzaramela/SynDNA), see notebook sequences_generation.ipynb. The synthetic sequences were selected following these criteria: (i) no self-complementary regions exceeding 15 bp (using SMS; https://www.bioinformatics.org/sms2/), (ii) covering a percentage of GC range from 26 to 66%, and (iii) addition of restriction enzyme site to the 5′ prime and 3′ prime ends. In addition, the optimized set of artificial sequences were submitted to analysis of sequence similarity using NCBI’s nt, est and est human nucleotide sequence databases (E value of <0.01; web-BLAST) to evaluate negligible identity to existing sequences. The synDNA sequences are listed in Table S2 in the supplemental material.

10.1128/msystems.00447-22.10 TABLE S2 synDNA sequences. Download Table S2, XLSX file, 0.06 MB. Copyright © 2022 Zaramela et al. 2022 Zaramela et al. https://creativecommons.org/licenses/by/4.0/ This content is distributed under the terms of the Creative Commons Attribution 4.0 International license.

### Plasmid synthesis and preparation.

The set of synDNAs were synthesized by GenScript Biotech using the GenPlus Next-Gen gene synthesis method (https://www.genscript.com/). The synDNAs were cloned into the plasmid pUC57 using plasmid standard preparation, and the constructs were lyophilized. Plasmids are available upon request at AddGene (IDs 186176 to 186185, see Addgene website: https://www.addgene.org/Karsten_Zengler/). Plasmids were cloned in E. coli DH5α. Qiagen maxiprep and miniprep purification kits were used to extract 10 plasmids containing the designed synDNAs from E. coli cells. To verify the presence of the synDNA in the plasmids, synDNAs were amplified by PCR using KAPA HiFi HotStart ReadyMix and visualized using 1% agarose gel.

### qPCR primer design and conditions.

Nonconserved regions for each of the 10 plasmids were selected using the NCBI genomic database. Primer pairs were designed for each plasmid using NCBI Primer-BLAST and IDT OligoAnalyzer with several parameters: 18- to 24-bp primer length, 150- to 200-bp product length, maximum melting temperature (*T_m_*) difference of 2°C, and 40 to 60% GC content. These parameters assume a general *T_m_* of 60 to 64°C and annealing temperature of 5°C below the *T_m_* of the primers. Additional characteristics were checked for when selecting the primer pair: secondary hairpin structures exhibit a *T_m_* more than 5 to 10°C below the annealing temperature, primer pairs have a *T_m_* within 5°C of each other, lower 3′ self-complementarity, no 3′ terminal thymine (T) base, and less than 3 GC bases at the 3′ position. Primers were diluted to 100 μM before a PCR was run using KAPA HiFi HotStart ReadyMix and visualized with 2% agarose gel to validate specificity via presence of a clearly unique band. The primers’ sequences are as follows: p126, forward, TCGAAGGCCATGCTGTGAAC, and reverse, GTTCGTGTACACTAGCAGTGATGA; p226, forward, GGATTAAATGCAGCGAGGTGTCA, and reverse, TTGCAACGGTCGTCATTGCTC; p136, forward, ATCGTTTGACCCTCCGCTCC, and reverse, TCCTGGAGTCTGTTCGCTTCA; p236, forward, TCTGGCACACGTCCAAGAGA, and reverse, GAAATGCTCAGCGTTGCGTG; p146, forward, AGTCGATGGTGCTGACTGGG, and reverse, AACTACAGAGTCGCCGGTCC; p246, forward, CCGGTTGAAGTCACGCCTTG, and reverse, CGCTCAAACCGCCTTACCAC; p156, forward, CTTTGCTTAGCCGCCGTCAG, and reverse, ATACCAGGCCAATCCCTCGC; p256, forward, CTCTAGGCCCGCGATTTCCA, and reverse, TACATGGCGTCGGTGCTTCA; p166, forward, CAAACGCTCTGTGACCTGGC, and reverse, TTGTGTGATGCGCGTGATCG; and p266, forward, GCGGTCGAATACCCTGCTGA, and reverse, ACCGGCAAGTCCCTATGAGC.

### Shotgun metagenomic libraries and sequencing.

Total genomic DNA was extracted using the ZymoBIOMICS DNA miniprep kit (Zymo Research; catalog no. D4300) following the manufacturer’s instructions. Total genomic DNA from human saliva samples was kindly provided by Clarisse Marotz (32). Total genomic DNA from the mock community was purchased from ZymoBIOMICS Microbial Community DNA (Zymo Research; catalog no. D6305). For both sample types (mock and saliva), the synDNA pools were mixed to the samples at 5% proportion before library prep. The 5% proportion of SynDNA concentration was chosen to obtain around 8% to 10% sequence coverage for the most diluted synDNA (0.0001 ng/μL), which corresponds to 100 to 150 reads from a 2-million-read sequencing run with a read length of 150 bp. Total DNA (biological sample plus synDNA pool) was prepared for shotgun metagenomic sequencing using the Nextera XT library preparation method, with an average fragment size of 2 × 150 bp (Illumina). Libraries were quality assessed using Qubit and Tapestation (Agilent Technologies) and subsequently sequenced using Novaseq 2 × 150-bp-cycle paired-end kit (Illumina). An average of 5 million reads were generated per library.

### Shotgun metagenomic data analysis.

Raw single-end or paired-end reads publicly available or generated in this study were initially trimmed and quality filtered using Trimmomatic (v. 0.39) with the following parameters (LEADING:10 TRAILING:10 SLIDINGWINDOW:4:15 MINLEN:36) (35). The reads from the publicly available data sets used to evaluate cross-alignment biases were trimmed to present the same length (50 bp). Trimmed reads were aligned to reference genomes, synDNA sequences (Table S2), and synthetic 16S rRNA gene sequences (17). The alignments were performed using Bowtie2 (v.2.3.2), with the flag “–very-sensitive” (36). To evaluate the synthetic 16S rRNA alignment bias, we used as references the genomes of Tourtoluse et al. (17) based on the design of the 16S synthetic rRNA gene: Escherichia coli ASM882318v1 (SRR10015223), Escherichia coli 9001_S21 (ERR1877982), Bacteroides vulgatus UBA7045 (SRR5327338), Bacteroides vulgatus (SRR8060827), Clostridium acetobutylicum RH8 (SRR1217786), Clostridium acetobutylicum ATCC824 (SRR1700598), Gemmatimonas aurantiaca MTG, Treponema bryantii NK4A124 (SRR896066), and Treponema bryantii NK4A124 (SRR4140225). In addition, to evaluate the occurrence of false-positive alignments to the synthetic 16S spike-in genes, we used the follow shotgun metagenomic data sets: human skin (PRJNA507269), human gut (PRJNA278393), mouse gut (PRJNA505660), human saliva (PRJEB24090), soil (PRJNA480881), and ocean (PRJNA385855, PRJNA488959) (21–26). For the Zymo mock community sequencing analysis, the reads were aligned to the reference genomes informed by the manufacturer. For the saliva sequencing analysis, the reads were aligned to the Human Microbiome Project (HMP) database (https://portal.hmpdacc.org/). The frequency tables were obtained using the Web of Life Toolkit app (Woltka) using the command “woltka classify” (37). To estimate the total number of cells per bacteria species, we used the genome length provided by Zymo Research or by the HMP reference table. To calculate the total number of cells per bacteria genus, when appropriate, we first calculate the number of cells at species level and add the total of number of cells per species from the same genus. Base call error rates were estimated following a similar strategy published by Tourtolouse et al. (17): in brief, an “NM” flag that indicates the number of mismatches per read was extracted from SAM files using a custom bash script (cat IN.sam | cut -d $'\t' -f 3,6,10,17 | sed 's/NM:i://' | awk -v OFS='\t' '{print$1,$2,length($3),$4}' > OUT.sam). To perform the analysis focused on reads aligned to Lactobacillus fermentum, Listeria monocytogenes, and Enterococcus faecalis genomes, the following custom bash script was applied: (cat IN.sam | grep -v “LN:\|@HD\|@PG”| grep “SPECIES”| cut -d $'\t' -f 3,6,10,17 | sed 's/NM:i://' | awk -v OFS='\t' '{print$1,$2,length($3),$4}' > OUT.sam). Plots and statistical analysis were generated using R scripts. Linear models were obtained using the function “*lm*,” package *stats*. Correlation analysis were performed using the functions “*cor*” and “*cor.test*,” package *stats*. Principal-component analyses (PCAs) were performed using the function “PCA,” package *FactoMiner*. Plots were obtained using the packages *ggplot2*, *factoextra*, and *graphics*. The code used to analyze the data can be found in https://github.com/lzaramela/SynDNA.

### Using synDNA linear models to predict the absolute number of cells.

We used the internal synDNA linear model (equation 1) to estimate the DNA weight per individual bacterial species based on the number of aligned reads to the same taxon

(1) $${\text{read weight}}_{i}={\text{intercept}}_{i}+\left({\text{slope}}_{i}\times X_{i}\right)$$

where *X_i_* represents the normalized number of reads, that is the total number of reads aligned to an individual bacterial species divided by the total number of sequenced reads.

To calculate the number of cells, we need to consider the DNA nucleotide base weight and the bacterial species genome size, as defined by equation 2:

(2) $$\text{no}{\text{. of cells}}_{i}=\frac{{\text{read weight}}_{i}\times 6.022\times 10^{23}}{Y_{i}\times 650}$$

where 6.022 × 10^23^ represents the Avogadro number, *Y_i_* represents the bacterial species genome length, and 650 represents the average molecular weight of a nucleotide base pair. The Avogadro number and the molecular weight of a nucleotide base pair are constants and have null effect when proportional compositions are being calculated.

Equation 1 and equation 2 allow the calculation of the total number of cells per each bacterial species and the elucidation of the real composition proportion among different taxa.

### Data availability.

Sequence data generated for this study are available at the Sequence Read Archive (SRA) under project no. PRJNA870099. synDNA sequences are listed in Table S2. The plasmids containing the synDNAs are available at Addgene under IDs 186176 to 186185. Code used to analyze the data can be found at https://github.com/lzaramela/SynDNA/.
