# Supplementary figures and images for: synDNA—a Synthetic DNA Spike-in Method for Absolute Quantification of Shotgun Metagenomic Sequencing
Source: mSystems. 2022 Nov 1;7(6):e00447-22. doi: 10.1128/msystems.00447-22 (PMC9765022; doi:10.1128/msystems.00447-22)

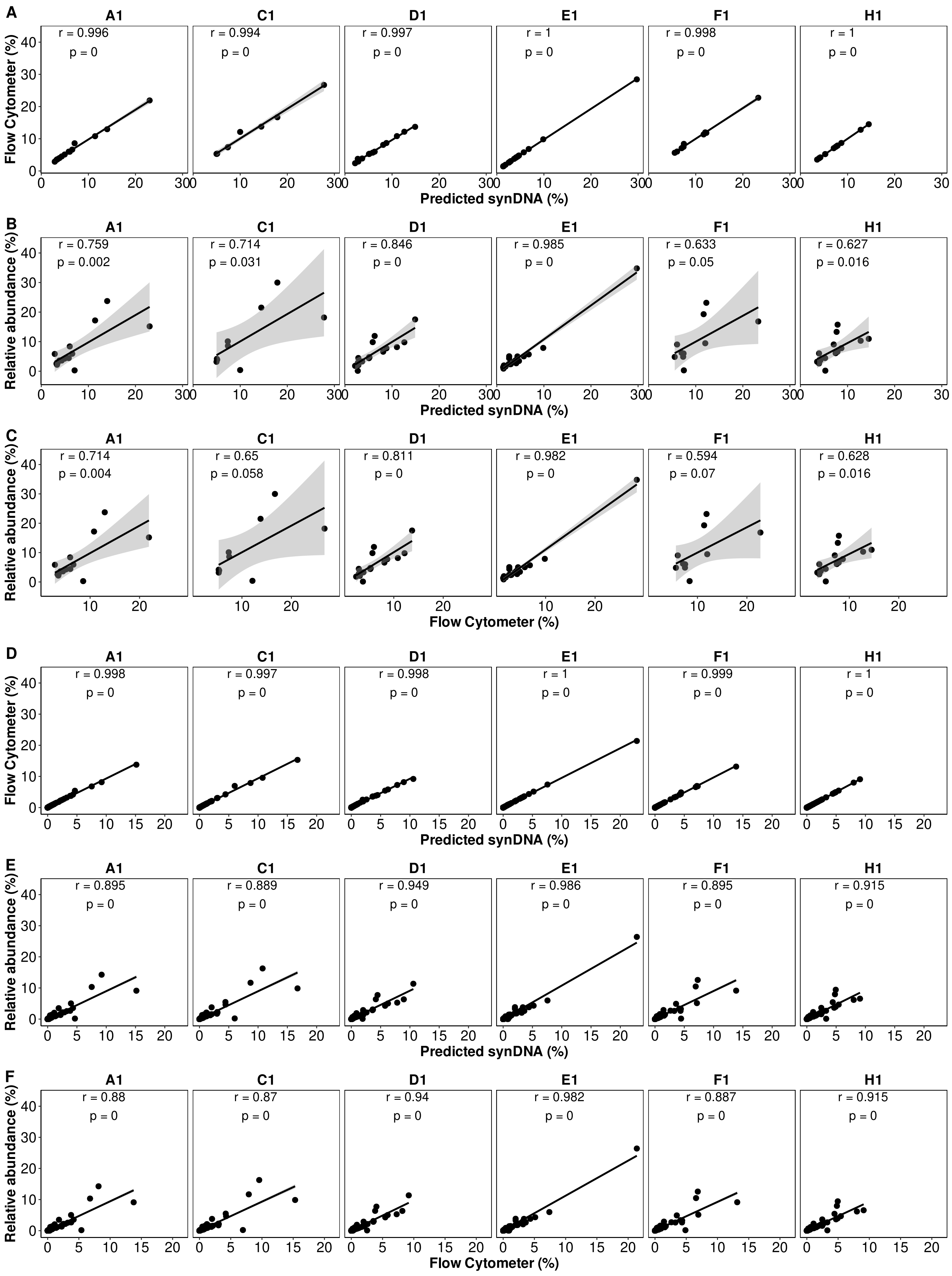

Supplement: FIG S8 [file msystems.00447-22-s0008.pdf]
